# Does molecular profiling of tumors using the Caris molecular intelligence platform improve outcomes for cancer patients?

**DOI:** 10.18632/oncotarget.24258

**Published:** 2018-01-16

**Authors:** Philip Carter, Costi Alifrangis, Biancastella Cereser, Pramodh Chandrasinghe, Lisa Del Bel Belluz, Thomas Herzog, Joel Levitan, Nina Moderau, Lee Schwartzberg, Neha Tabassum, Jinrui Wen, Jonathan Krell, Justin Stebbing

**Affiliations:** ^1^ Department of Surgery and Cancer, Imperial College, London, UK; ^2^ Department of Oncology, University College Hospital, London, UK; ^3^ Department of Surgery, University of Kelaniya, Kelaniya, Sri Lanka; ^4^ Department of Obstetrics and Gynecology, University of Cincinnati, Cincinnati, USA; ^5^ University of Cincinnati Cancer Institute, University of Cincinnati, Cincinnati, USA; ^6^ WEST Cancer Center, The University of Tennessee, Memphis, USA

**Keywords:** tumor molecular profiling, cancer treatment

## Abstract

We evaluated the effect of tailoring treatments based on predictions informed by tumor molecular profiles across a range of cancers, using data from Caris Life Sciences. These included breast carcinoma, colorectal adenocarcinoma, female genital tract malignancy, lung non-small cell lung cancer, neuroendocrine tumors, ovarian surface epithelial carcinomas, and urinary tract cancers.

Molecular profiles using mostly immunohistochemistry (IHC) and DNA sequencing for tumors from 841 patients had been previously used to recommend treatments; some physicians followed the suggestions completely while some did not. This information was assessed to find out if the outcome was better for the patients where their received drugs matched recommendations.

The IHC biomarker for the progesterone receptor and for the androgen receptor were found to be most prognostic for survival overall. The IHC biomarkers for P-glycoprotein (PGP), tyrosine-protein kinase Met (cMET) and the DNA excision repair protein ERCC1 were also shown to be significant predictors of outcome. Patients whose treatments matched those predicted to be of benefit survived for an average of 512 days, compared to 468 days for those that did not (*P* = 0.0684). In the matched treatment group, 34% of patients were deceased at the completion of monitoring, whereas this was 47% in the unmatched group (*P* = 0.0001).

## INTRODUCTION

Cancer is a disease of tissue growth deregulation that causes approximately 15% of deaths globally, which was about 8 million in 2012 [1, 2]. Germline mutations drive only around 1 percent of tumors [3]. Multistep tumorigenesis occurs through consecutive changes, that include mutations in oncogenes, epigenetic silencing of tumor suppressor genes, and chromosomal aberrations. These changes underlie the causes of cancer, along with heterotypic interactions between the tumor and its microenvironment, conferring a selective advantage leading to clonal expansion. A typical breast or colon carcinoma will have 60 to 70 mutations, of which about three or four may drive this expansion, while the others may be viewed as passengers [4].

Guided treatments via molecular characterization of solid tumors using biomarkers such as immunohistochemistry (IHC) and genomic sequencing has resulted in better outcomes in subsets of patients, such as those with *EGFR* (epidermal growth factor receptor) mutant lung cancer and *BRAF* mutant melanoma [5]. Many organizations are now providing molecular profiling platforms to clinicians to guide therapeutic decisions in the context of metastatic and early disease. Platforms such as Oncotype DX, Foundation One, EndoPredict and Caris Life Sciences’ Molecular Intelligence are commercially available, and are used in routine clinical practice in the UK and the USA. Elsewhere, the American Society of Clinical Oncology (ASCO) launched CancerLinQ in 2015, which uses records from thousands of oncology treatment centers to improve patient care, by recommending treatment plans using this information. The Oncology Research Information Exchange Network (ORIEN), which has been initiated by the Moffitt Cancer Center, uses clinical data and tissue samples in a similar way. IBM’s Watson Health is also using clinical data to aid the design of cancer therapy plans.

Here we used data from Caris Life Sciences to see if tumor molecular profiling led to better clinical of outcome when used to give treatment recommendations.

## RESULTS

### Patient characteristics

Data describing clinical outcomes for patients with a range of cancer types was assessed to see if there was a benefit when their treatment regimens adhered to recommendations that utilized tumor molecular profiling to select drugs. In the matched treatment set, patients were given at least one recommended drug after collection for tumor profiling and none that were not. In the unmatched set one or more drugs that were predicted to lack benefit were in the treatment plan. The matched group consisted of 438 patients and the unmatched 403 patients. Patients and their primary tumors are summarized in Tables 1 and 2.

**Table 1:** Cancer types in the Caris data

Cohort	Matched	Unmatched	Total
Adrenal cortical carcinoma	-	1	1
Anal cancer	1	3	4
Breast carcinoma	43	49	92
Cholangiocarcinoma	5	1	6
Colorectal adenocarcinoma	42	53	95
CUP	1	8	9
Epithelial skin cancer	1	1	2
Female genital tract malignancy	64	48	112
Gastroesophageal adenocarcinoma	8	15	23
Glioblastoma	4	1	5
Head and neck squamous carcinoma	5	6	11
Leiomyosarcoma	9	5	14
Liver hepatocellular carcinoma	2	1	3
Lung bronchioloalveolar carcinoma (BAC)	-	1	1
Lung non-small-cell lung cancer (NSCLC)	42	49	91
Lymphoma	2	-	2
Major and minor salivary glands	3	-	3
Melanoma	3	4	7
Neuroblastoma	-	1	1
Neuroendocrine tumors	13	10	23
Non epithelial ovarian cancer (non EOC)	2	2	4
Ovarian surface epithelial carcinomas	154	115	269
Pancreatic adenocarcinoma	7	9	16
Paragangliomas	1	-	1
Prostatic adenocarcinoma	2	1	3
Small intestinal malignancies	-	1	1
Soft tissue tumors	7	10	17
Urinary tract	16	8	24
Uveal melanoma	1	-	1

Cancer cohorts present in the Caris data used here, the number of patients that are present in them, and how each cohort can be divided into patients whose treatments matched those that were recommended based on a molecular profile of their tumor, and those whose did not

**Table 2:** Patient ages for the matched and unmatched treatment groups

Age	Matched	Unmatched
20–29	3	7
30–39	16	11
40–49	54	51
50–59	111	105
60–69	130	124
70–79	106	81
80–89	18	24

### Treatment analysis

**Figure 1:**
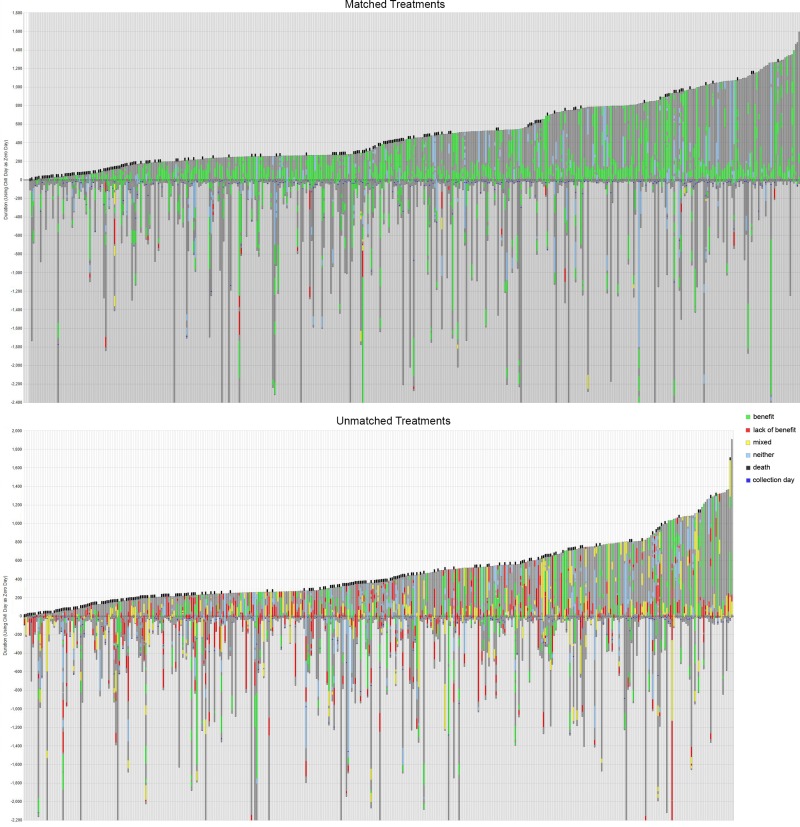
Matched and unmatched treatment groups’ drug schedules and their outcomes Shown above are treatment plans for 438 matched patients, and in the lower plot treatment plans for 403 unmatched patients. The y-axis is time in days and zero is the time of tumor profiling, so that they are ordered by increasing post-profiling survival time. Dark gray is total time monitored from diagnosis, and this ends either in last follow-up or when the patient is deceased; a black line at the top of a column indicates death. Green is time on a treatment expected to be of benefit, red is treatment expected to lack benefit, and yellow is a combined therapy that uses drugs profiled to be both of benefit and to lack benefit. Blue is a neutral therapy that has neither associated prediction of benefit nor lack of benefit. Please note that a small number of treatment plans are not shown from their start (i.e. are incomplete at their base) for the purpose of brevity; the maximum time before collection for profiling was 11,673 days.

The individual treatments for the patients are shown for the two groups in Figure 1. There are 438 matched and 403 unmatched patients represented by a vertical line for each. Green shows drugs of benefit, red is a drug that lacks benefit, and yellow is both of these types of drug at the same time.

Table 3 shows the frequency of drugs given. The number of patients treated with a drug is shown in the first column, and the number of continuous treatment periods is shown in all other columns, i.e. treatments of the same patient with intervening periods are counted separately. The drugs given to the most number of patients were carboplatin (513 patients), paclitaxel (425), bevacizumab (215), gemcitabine hydrochloride (203), docetaxel (196), and cisplatin (186). The most common drugs by counting continuous treatments were carboplatin (given for 701 periods of time), paclitaxel (556), bevacizumab (310), fluorouracil (242), gemcitabine hydrochloride (241), docetaxel (223), and cisplatin (214 times).

**Table 3:** Drug frequencies

Number of Patients Treated	Most Frequently Administered Drugs (Total Treatment Periods)							
All Patients Treated	All Patients – Treatment Periods	Matched Only Patients, All Treatments	Matched, After Profiling Treatments Only	Unmatched Patients, All Treatments	Unmatched, After Profiling Treatments Only	Drugs Predicted of Benefit	Drugs Predicted to Lack Benefit	Drugs with No Prediction (Neither of Benefit or Lack of Benefit)
carboplatin – 513 patients	carboplatin = 701	carboplatin = 361	carboplatin = 166	carboplatin = 340	carboplatin = 120	carboplatin = 339	carboplatin = 162	bevacizumab = 235
paclitaxel – 425 patients	paclitaxel = 556	paclitaxel = 291	paclitaxel = 129	paclitaxel = 265	bevacizumab = 92	paclitaxel = 326	paclitaxel = 93	carboplatin = 194
bevacizumab – 215 patients	bevacizumab = 310	bevacizumab = 119	bevacizumab = 69	bevacizumab = 191	paclitaxel = 90	fluorouracil = 160	gemcitabine hydrochloride = 67	leucovorin calcium = 148
gemcitabine hydrochloride – 203 patients	fluorouracil = 242	docetaxel = 118	docetaxel = 49	fluorouracil = 168	gemcitabine hydrochloride = 79	gemcitabine hydrochloride = 112	cisplatin = 61	paclitaxel = 129
docetaxel – 196 patients	gemcitabine hydrochloride = 241	gemcitabine hydrochloride = 101	gemcitabine hydrochloride = 48	gemcitabine hydrochloride = 140	fluorouracil = 68	docetaxel = 108	doxorubicin hydrochloride = 53	cyclophosphamide = 103
cisplatin – 186 patients	docetaxel = 223	cisplatin = 100	cisplatin = 40	cisplatin = 114	cisplatin = 45; leucovorin calcium = 45	cisplatin = 104	fluorouracil = 45	docetaxel = 67
fluorouracil – 143 patients	cisplatin = 214	fluorouracil = 74	capectabine = 25; fluorouracil = 25	leucovorin calcium = 106	-	doxorubicin hydrochloride = 79	docetaxel = 44	gemcitabine hydrochloride = 59
doxorubicin hydrochloride – 136 patients	leucovorin calcium = 156	doxorubicin hydrochloride = 72	-	docetaxel = 105	docetaxel = 39	bevacizumab = 68	irinotecan hydrochloride = 43	capecitabine = 50
oxaliplatin – 116 patients	doxorubicin hydrochloride = 144	oxaliplatin = 55	doxorubicin hydrochloride = 24	oxaliplatin = 91	irinotecan hydrochloride = 35	pegylated liposomal doxorubicin hydrochloride = 67	Oxaliplatin = 38	cisplatin = 46
cyclophosphamide – 101 patients	oxaliplatin = 141	leucovorin calcium = 50	pegylated liposomal doxorubicin hydrochloride = 22	doxorubicin hydrochloride = 74	pegylated liposomal doxorubicin hydrochloride = 32	oxaliplatin = 64	topotecan hydrochloride = 18	oxaliplatin = 38

Drugs given most often in the matched and unmatched treatment groups and those given most frequently that were profiled to be beneficial, lacking benefit, or neither.

(Number of patients that received a drug is shown in the first column; number of treatments i.e. continuous periods of receiving a drug is shown in all other columns).

On average patients received 4.8 drugs (counting separate treatment periods). Of these, 45% were of benefit, 19% lacked benefit, and 36% neither of these types. Matched patients had 4.2 treatments on average – 60% of these were expected to be of benefit, 3% lacked benefit. Unmatched patients had an average of 5.5 drugs; 32% of these were of benefit, 33% lacking benefit.

The drugs of benefit given most often were carboplatin (339 treatments), paclitaxel (326), fluorouracil (160), gemcitabine hydrochloride (112), docetaxel (108), and cisplatin (104).

The treatments that were profiled to lack benefit given most often were carboplatin (162 periods of time), paclitaxel (93), gemcitabine hydrochloride (67), and cisplatin (61).

In the matched group 37% had no associated recommendation i.e. neither of type benefit or lack of benefit, and 35% in the unmatched group. The most popular in the neither category was bevacizumab (given 235 times), followed by carboplatin (194), leucovorin calcium (148), paclitaxel (129), and cyclophosphamide (103).

We identified off-label use of ER, PR & AR associated therapies, but this only occurred in two of the 841 patients; although they had 100% survival, it is not possible to draw conclusions from this due to the small number of patients.

We looked for potentially actionable mutations that were in the tumour profile, and corresponding treatments, using the following types.

BRAF mutations in melanoma, colorectal, NSCLC and cholangiocarcinoma cohorts, with treatment using sorafenib tosylate or regorafenib.

EGFR mutations in NSCLC and glioblastomas, treated with erlotinib hydrochloride, afatinib dimaleate, or lapatinib ditosylate.

ERBB2 mutations in breast, ovarian, NSCLC and gastroesophageal, with afatinib dimaleate or lapatinib ditosylate.

FGFR1 mutations in lymphoma using regorafenib or pazopanib hydrochloride.

FGFR2 mutations in gastroesophageal, NSCLC or female genital tract with regorafenib.

PDGFRA mutations in glioblastoma, treated with regorafenib, sunitinib malate or pazopanib hydrochloride.

RET mutations in adrenal cortical carcinoma or NSCLC, using cabozantinib-s-malate, sorafenib tosylate, regorafenib or sunitinib malate.

ALK mutations in NSCLC treated with crizotinib.

From this, we found 67 examples of clearly actionable mutations in 59 patients (35 were unmatched treatment patients, 24 were matched). There were at most two actionable mutations in any patient. Nine patients out of the 59 (15%) actually received a therapy clearly specific to the mutation; these all used regorafenib to treat colorectal patients with BRAF mutations. These nine patients had a mortality rate of 55%, an average survival after diagnosis (include those that died and those that were still alive when records finished) of 1202.7 days, and a post-profiling time on record of 385.7 days. The mortality rate here is worse than the colorectal cohort’s average, as is the time of monitoring after profiling, but the time of recorded monitoring after diagnosis is longer than the average.

**Figure 2:**
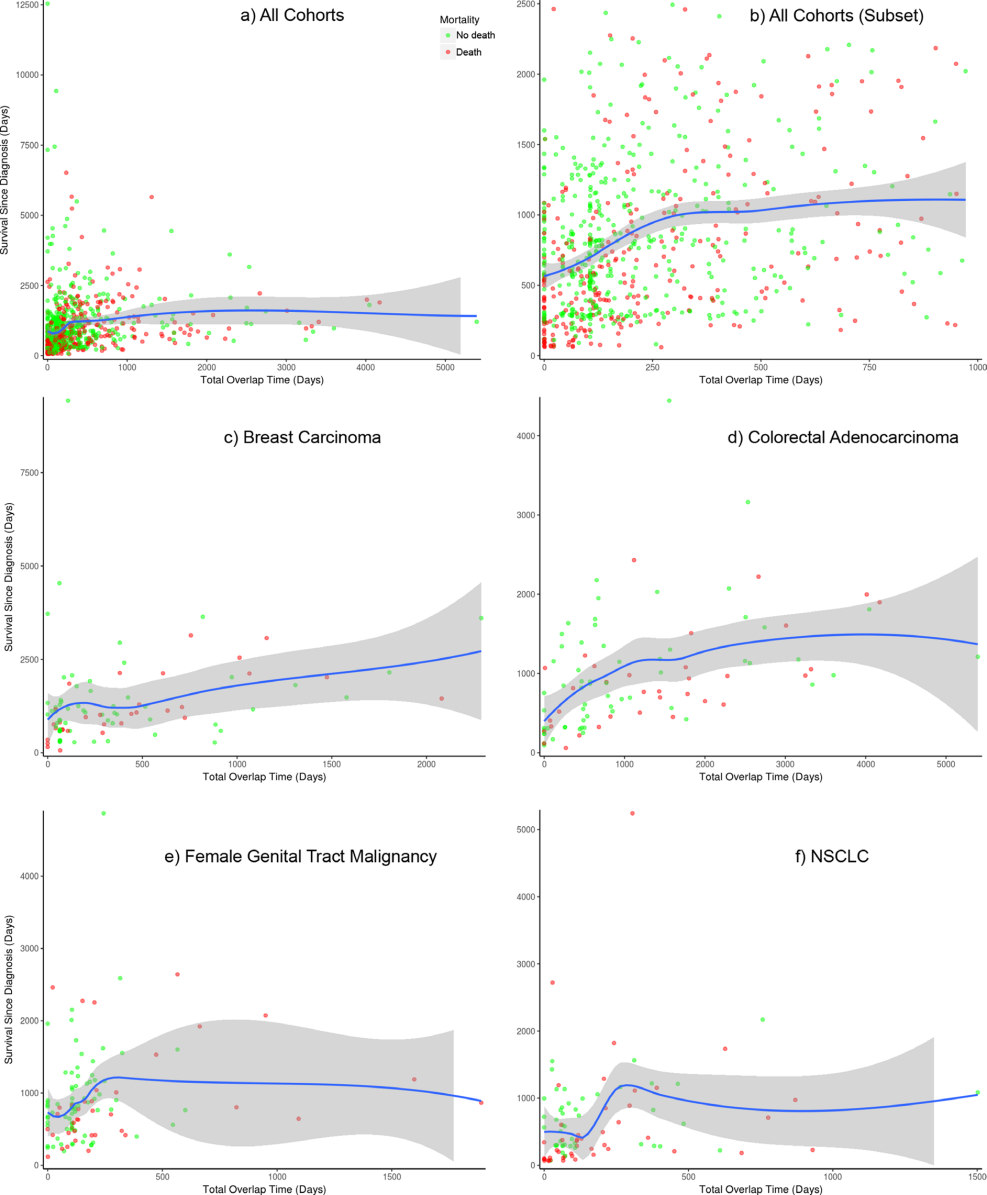
Comparison of survival with number of overlapping treatment days Plots for (**A**) all cohorts combined of which (**B**) zooms into the lower-left of it, and (**C**) breast carcinoma, (**D**) colorectal adenocarcinoma, (**E**) female genital tract malignancy and (**F**) NSCLC cohorts. Days were counted for each pair of overlapping treatments. The blue line is a loess curve that combines linear least squares regression with nonlinear regression to provide a smooth curve through the points. The gray band surrounding the blue curve is it’s 95% confidence interval. Points are colored to represent mortality: red denotes death of the patient, green represents no death by the end of monitoring.

We investigated if there was an improvement in outcome when treatments occur at the same time, irrespective of whether they were categorized as matched or unmatched. Figure 2 shows plots for the change in survival when overlapping treatment days increases (days were counted for each pair of overlapping treatments in each patient, so that for example, if three drugs were given at the same time, the overlap time was counted twice). We assessed the “all cohorts combined” dataset, and also the breast, colorectal, female genital tract malignancy and NSCLC cohorts separately. There does appear to be an improvement overall when giving treatments simultaneously for breast and colorectal, as shown in Figure 2. For the female genital tract malignancy and NSCLC cohorts, there is an improvement as the amount of time that drugs are given concurrently increases, but this later decreases, although this is outside the main body of points that each represent a patient. This is also the case in the all cohorts combined plot shown in Figure 2A) i.e. an initial improvement tails off and then slightly decreases for a small number of patients with the highest total overlap times. The upper-right plot, Figure 2B), shows a subset of patients from the all cohorts dataset, restricting to those with total overlap time of 1000 days or less, and 2500 days survival after diagnosis or less, so as to zoom in on the lower-left of the plot shown in Figure 2A). We further analyzed the data to see if this could be explained by categorizing the overlapping treatments, using a variety of classes such as chemotherapy, hormonal therapies, different types of targeted therapies excluding monoclonal antibodies (e.g. kinase inhibitors, etc.), monoclonal antibodies, and others. However, we did not find a clearly better way of combining therapies when looking at the outcome data in this way. Similarly, we did not find a distinctly better sequence of treatments i.e. where one class of therapy was given before another.

### Patient survival

**Figure 3:**
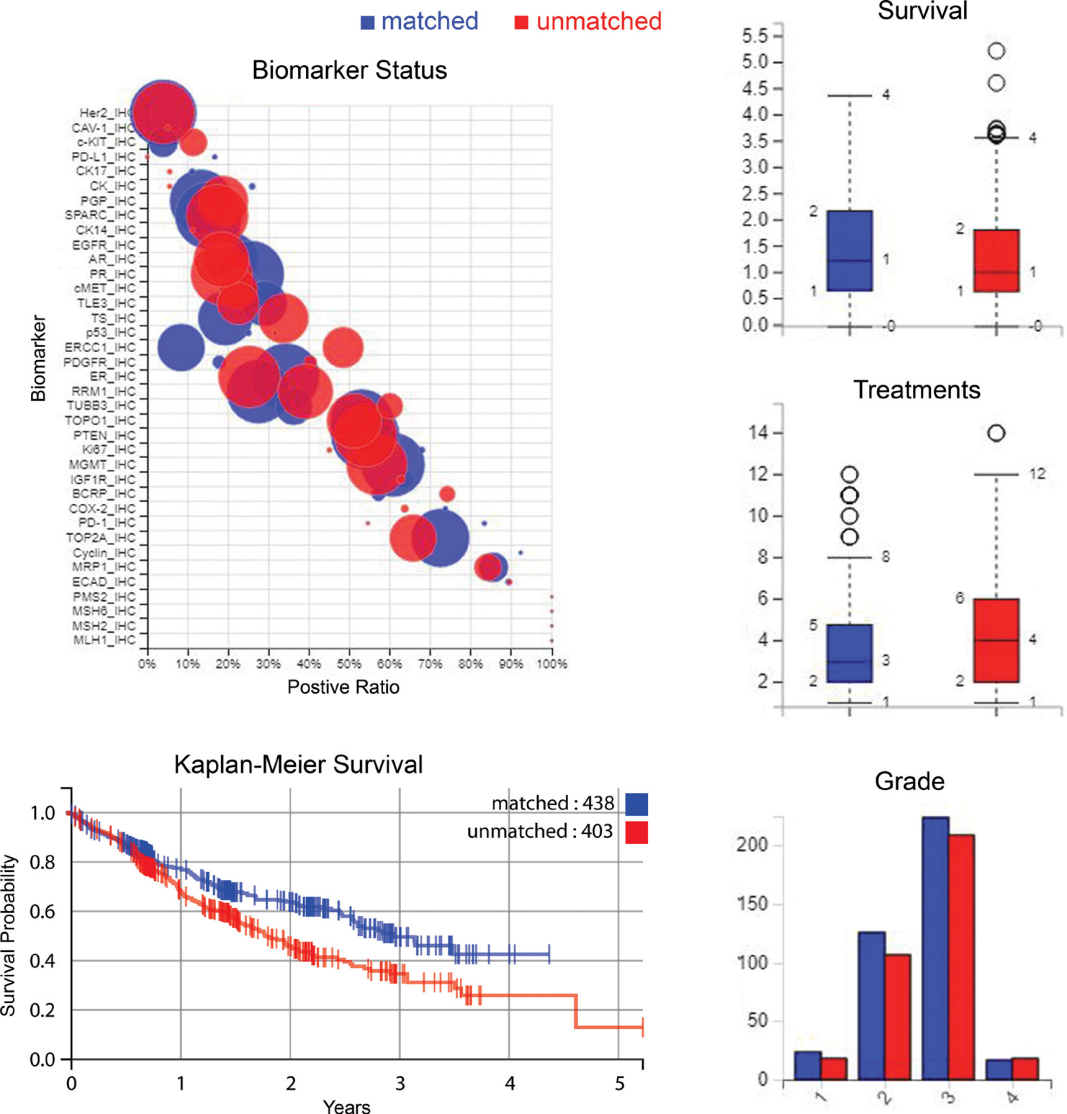
Biomarker statuses, patient survival, number of treatments and tumor grades Clockwise from *upper-left*: Status of biomarkers contrasted between the matched and unmatched treatment groups where “positive ratio” is the percentage that have positive biomarker results - positive indicates protein expression above a predefined threshold in IHC markers for example, and for sequencing markers positive denotes a gene mutation that is usually pathogenic. The size of the circle indicates the number of cases. *Upper-right to lower-right*: survival time, treatment numbers, and grade of samples are summarized. *Lower-left*: a Kaplan-Meier plot of survival time for patients treated with recommended therapies only, compared to patients who had at least one therapy that had been predicted to have no benefit i.e. matched versus unmatched.

In the matched group 34% of patients died compared to 47% of the unmatched group of patients. Figure 3 shows a Kaplan-Meier curve of overall survival for patients treated only with therapies predicted to be beneficial, compared to those of the unmatched treatment group. Figure 3 also shows biomarker differences between the matched and unmatched groups, and summaries of survival, treatment and sample information. Table 4 shows a summary of the differences in survival between some of the larger cancer categories available in the data analyzed here, along with biomarkers and drugs given most often for those cancer subsets.

**Table 4:** Comparison of some of the major cohorts in the Caris CODE database

Cohort	Total Number of Patients (Matched Patients; Unmatched Patients)	Post Profiling Survival in Days – Matched vs Unmatched	Mortality – Matched vs Unmatched	Significant Biomarkers	Most Frequent Drugs
Breast	92 (43; 49)	667 vs 510 (*P* = 0.03)	26% vs 41% (*P* = 0.13)	AR, ER, PR	cyclophosphamide, doxorubicin hydrochloride, docetaxel
Colorectal	95 (42; 53)	442 vs 541 (*P* = 0.1773)	19% vs 49% (*P* = 0.0022)	TS (thymidylate synthase)	fluorouracil, leucovorin calcium, oxaliplatin
Female genital tract	112 (64; 48)	593 vs 449 (*P* = 0.03)	30% vs 40% (*P* = 0.28)	PR	carboplatin, paclitaxel, cisplatin
Lung	91 (42; 49)	402 vs 382 (*P* = 0.79)	48% vs 53% (*P* = 0.61)	ERCC1, EGFR	carboplatin, pemetrexed disodium, docetaxel
All cancers combined (including others)	841 (438; 403)	512 vs 468 (*P* = 0.07)	34% vs 47% (*P* = 0.0001)	PR, AR, PGP (P-glycoportein), cMET, ERCC1	carboplatin, paclitaxel, bevacizumab

### Predictive biomarkers

**Figure 4:**
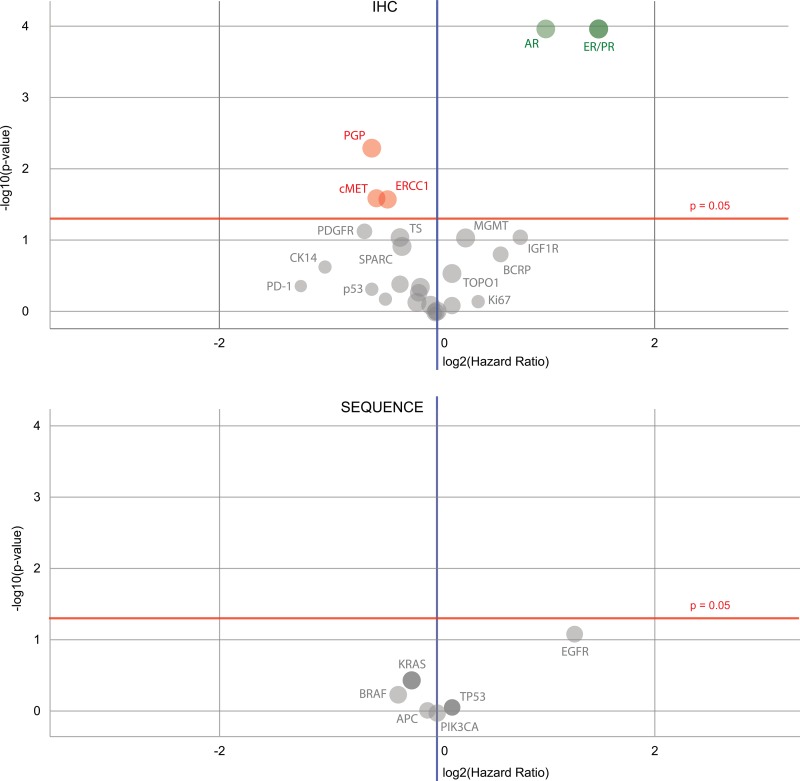
Volcano plots of biomarkers’ prognostic values In the plot shown above, several IHC biomarkers are of significance – ER, PR, AR (all are on the top right in green), also PGP, cMET and ERCC1 (in red) – and in the plot shown below, the EGFR sequencing marker stands out from the others, but is not statistically significant. In both plots, green dots indicate when the hazard rate of a positive biomarker result is significantly lower than that of a negative biomarker result, whereas a red dot is for when the hazard rate of a positive biomarker result is significantly higher than that of a negative biomarker result; gray dots denote when the difference between a positive and negative biomarker result is not significant.

**Figure 5:**
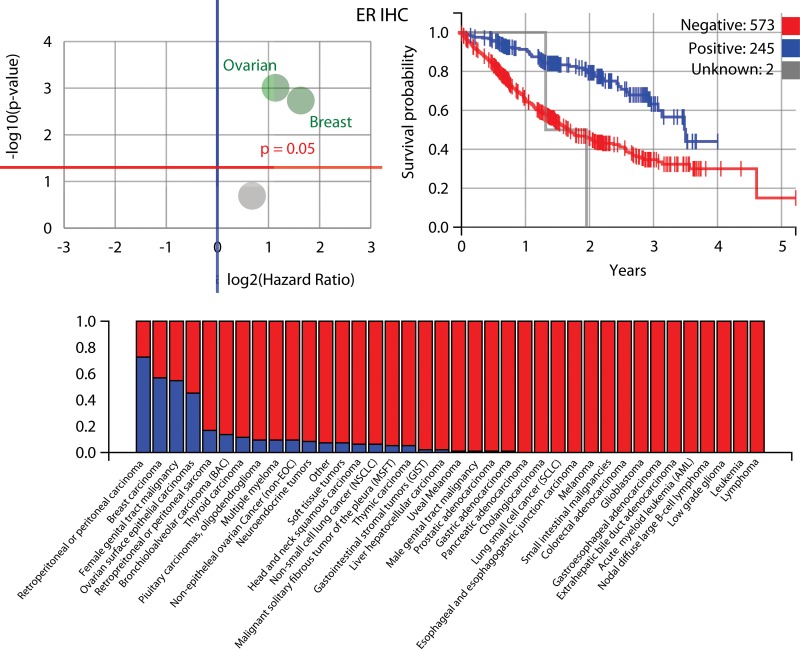
Further information about the estrogen receptor (ER) IHC biomarker, which was identified to be most prognostic for patient outcomes along with PR and AR *Upper left*: ER is highly prognostic for ovarian surface epithelial cancer and breast cancer, as shown in this volcano plot. *Upper right*: a Kaplan-Meier plot for ER, i.e. those patients with a positive biomarker result have an improved outcome compared to those that do not, although this lessens in the long-term. *Below*: positive ratio for the ER IHC biomarker in different cancer cohorts within the Caris data i.e. the percentage in each cohort that have a positive biomarker result, when measuring protein expression above a predefined threshold (blue is positive).

**Figure 6:**
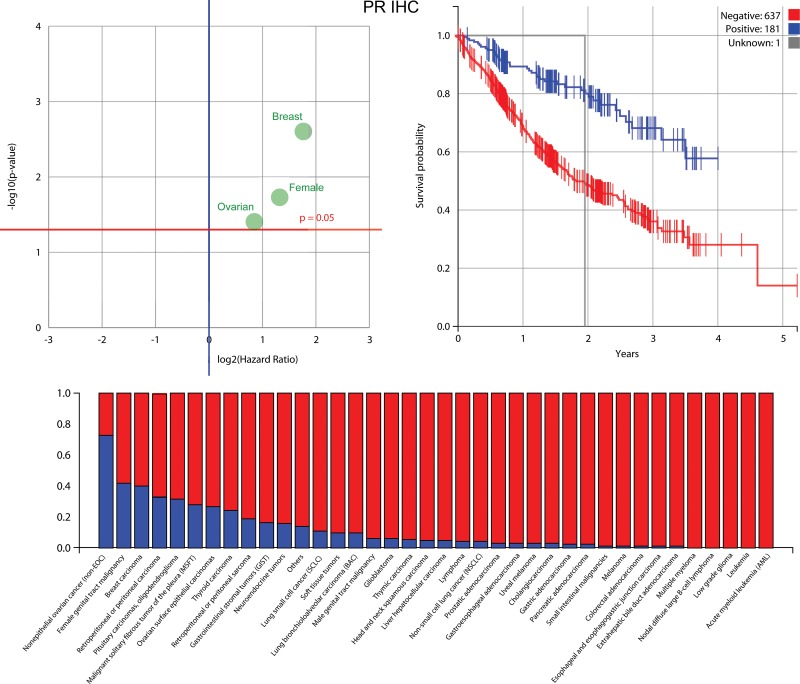
Information about the progesterone receptor (PR) biomarker, which was found to be most prognostic for patient outcomes along with ER and AR *Upper left*: PR is highly prognostic for ovarian surface epithelial cancer, breast cancer and female genital tract malignancy. *Upper right*: a Kaplan-Meier plot for PR that shows that patients with a positive biomarker result have an improved outcome. *Below*: the percentage in each cohort that have a positive PR biomarker result (blue is positive).

**Figure 7:**
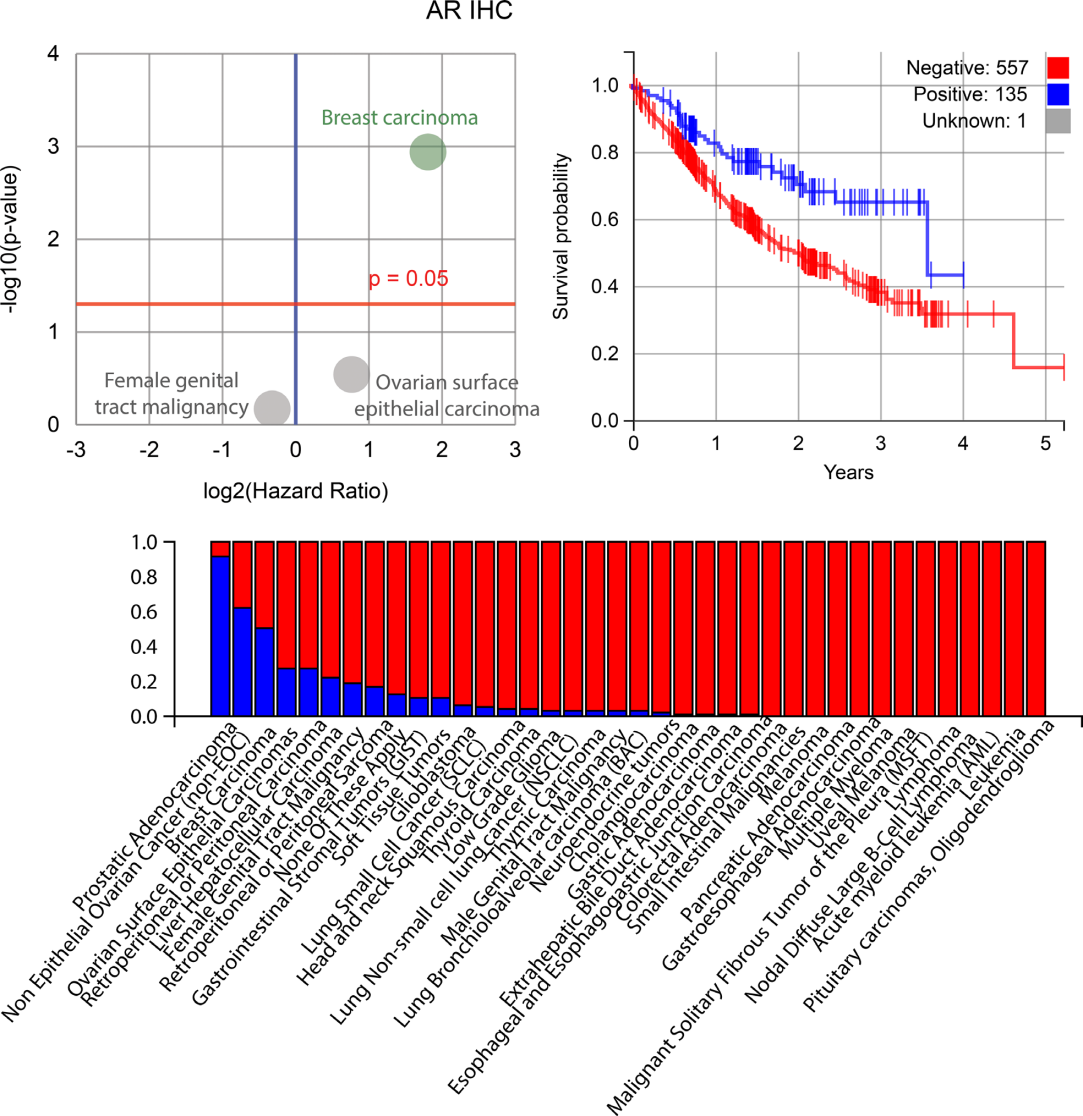
Plots for the androgen receptor (AR) biomarker, which was indicated to be significantly prognostic for patient outcomes along with ER and PR *Upper left*: AR is highly prognostic for breast cancer, as shown in this volcano plot. *Upper right*: a Kaplan-Meier plot for PR where patients positive for AR have an improved outcome, although this narrows over the long-term. *Below*: the percentage in each cohort that have a positive AR biomarker result (blue is positive).

The IHC biomarkers that were good predictors for survival (Figure 4) were the estrogen receptor (ER) (in ovarian surface epithelial carcinomas and breast carcinomas), progesterone receptor (PR) (in breast carcinomas, female genital tract malignancies, and ovarian surface epithelial carcinomas) and androgen receptor (AR) (in breast carcinoma), along with those for P-glycoprotein (PGP) (no specific cohort), tyrosine-protein kinase Met (cMET) (no specifc cohort) and the DNA excision repair protein ERCC1 (in NSCLC). The best sequencing biomarker was for the epidermal growth factor receptor gene (in NSCLC), but it was less significant (*P* = 0.0764) than the best IHC markers. Further information about the biomarkers identified to be most prognostic for patient survival is shown in Figures 5, 6 and 7 (ER, PR and AR IHCs respectively). Although they did not stand out as significant across the background of all cohorts, TOPO1 was a significant predictor of outcome within the ovarian patients, as was TS (thymidylate synthase) for colorectal patients. If breast cancer patients are removed from the set of all other patients analysed here, ER and PR are still significant predictors of importance, but AR is no longer a significant predictor of outcome.

## DISCUSSION

This report looked at clinical outcomes for a range of tumor type cohorts that were profiled by Caris Life Sciences to predict treatments using an algorithm. Treatments after tumor profiling were classed as matched or unmatched, according to if the drugs given by their doctor agreed with the recommendations that had utilized the molecular profile of their tumor.

The unmatched group received more treatments in general than the matched group, and had a poorer survival prognosis. This could have been due to the unmatched group having tumors that were more advanced in stage than in the matched group, as shown in Table 5.

**Table 5:** Matched and unmatched groups compared against all

Group	Average Age	Ethnicity	Grade	Stage	Survival (Days)	Mortality
All patients (841)	61.5	White: 721; Black/African American: 70; Asian: 25; Hawaiian/Pacific Islander: 11; Other/unknown: 11; American Indian/Alaskan Native: 3	Grade 4/Undifferentiated: 35 (4%); Grade 3/Poorly differentiated: 433 (51%); Grade 2/Moderately differentiated: 232 (28%); Grade 1/Well differentiated: 41 (5%); Unknown/Not determined: 77 (9%); None/Not applicable: 16 (2%); High Grade: 6 (1%); Low Grade: 1 (−%)	IV: 223 (27%); III no IIIC: 136 (16%); IIIC: 180 (21%); II: 134 (16%); I: 100 (12%); Unknown: 68 (8%)	491	40%
Matched only (438)	61.6	White: 372; Black/African American: 33; Asian: 16; Hawaiian/Pacific Islander: 8; Other/unknown: 8; American Indian/Alaskan Native: 1	Grade 4/Undifferentiated: 17 (4%); Grade 3/Poorly differentiated: 224 (51%); Grade 2/Moderately differentiated: 125 (29%); Grade 1/Well differentiated: 23 (5%); Unknown/Not determined: 39 (9%); None/Not applicable: 7 (2%); High Grade: 2 (−%); Low Grade: 1 (−%)	IV: 102 (23%); III no IIIC: 74 (17%); IIIC: 95 (22%); II: 69 (16%); I: 60 (14%); Unknown: 38 (8%)	512	34%
Unmatched (403)	61.3	White: 349; Black/African American: 37; Asian: 9; Hawaiian/Pacific Islander: 3; Other/unknown: 3; American Indian/Alaskan Native: 2	Grade 4/Undifferentiated: 18 (4%); Grade 3/Poorly differentiated: 209 (52%); Grade 2/Moderately differentiated: 107 (27%); Grade 1/Well differentiated: 18 (4%); Unknown/Not determined: 38 (10%); None/Not applicable: 9 (2%); High Grade: 4 (1%)	IV: 121 (30%); III no IIIC: 62 (15%); IIIC: 85 (21%); II: 65 (16%); I: 40 (10%); Unknown: 30 (8%)	468	47%

The survival curves diverge after the time of profiling (Figure 3), which could indicate that therapy predictions had an effect on improving outcome, due to selection of optimal therapies. We see three IHC biomarkers being prognostic for poorer outcomes across the cohort (PGP, cMET and ERCC1) and suggest that these are further evaluated in prospective cohorts. Combined with the reduction in mortality, this indicates that there is a benefit from tumor molecular profiling here. This may be that part of this is due to the profiles uncovering the group of ER, PR and AR positive tumors that are generally associated with a better outcome.

We also found that the more time that patients received overlapping therapies, the more their survival time improved, in general.

## MATERIALS AND METHODS

The Caris CODE database contains tumor molecular profile data for 841 patients with solid tumors (CODE version 1.0). It also includes demographic information about these patients, the drug treatments that they received before and after molecular profiling, and records of their clinical outcomes while they were still being monitored. This data was mined after web scraping the data from the Caris website, to understand if molecular characterization affected drug selection by treating physicians, and if any subtypes of molecular subsets had different outcomes across tumor types. Tables 1 and 2 describe the clinical characteristics of the patients that were profiled.

The amount of time that patients were monitored varied, as shown in Figure 1. On average patients’ treatment records were available for 1018 days after diagnosis (1034 days for matched treatment patients and 1001 days for unmatched patients). On average the time of monitoring after profiling was 491 days, and the longest period of monitoring after profiling was 1906 days (the patient represented on the furthest right of Figure 1); this was 1920 days after diagnosis. The longest that records were available for any patient i.e. from diagnosis up until the last day of contact, was 12,537 days.

## Abbreviations

ASCO: American Society of Clinical Oncology
CODE: Comprehensive Oncology Database Explorer
EGFR: epidermal growth factor receptor
IHC: immunohistochemistry
ER: estrogen receptor
ORIEN: Oncology Research Information Exchange Network
PGP: P-glycoprotein
PR: progesterone receptor
cMET: tyrosine-protein kinase Met
