# Correction: Does molecular profiling of tumors using the Caris molecular intelligence platform improve outcomes for cancer patients?

**DOI:** 10.18632/oncotarget.24691

**Published:** 2018-03-13

**Authors:** Philip Carter, Costi Alifrangis, Biancastella Cereser, Pramodh Chandrasinghe, Lisa Del Bel Belluz, Thomas Herzog, Joel Levitan, Nina Moderau, Lee Schwartzberg, Neha Tabassum, Jinrui Wen, Jonathan Krell, Justin Stebbing

**Affiliations:** ^1^ Department of Surgery and Cancer, Imperial College, London, UK; ^2^ Department of Oncology, University College Hospital, London, UK; ^3^ Department of Surgery, University of Kelaniya, Kelaniya, Sri Lanka; ^4^ Department of Obstetrics and Gynecology, University of Cincinnati, Cincinnati, USA; ^5^ University of Cincinnati Cancer Institute, University of Cincinnati, Cincinnati, USA; ^6^ WEST Cancer Center, The University of Tennessee, Memphis, USA

**This article has been corrected:** The proper Materials and Methods and Conflicts of Interest information is as follows:

**MATERIALS AND METHODS**

The Caris CODE database contains tumor molecular profile data for 841 patients with solid tumors (CODE version 1.0). It also includes demographic information about these patients, the drug treatments that they received before and after molecular profiling, and records of their clinical outcomes while they were still being monitored. This data was mined after web scraping the data from the Caris website, to understand if molecular characterization affected drug selection by treating physicians, and if any subtypes of molecular subsets had different outcomes across tumor types. Tables 1 and 2 describe the clinical characteristics of the patients that were profiled.

The amount of time that patients were monitored varied, as shown in Figure 1. On average patients’ treatment records were available for 1018 days after diagnosis (1034 days for matched treatment patients and 1001 days for unmatched patients). On average the time of monitoring after profiling was 491 days, and the longest period of monitoring after profiling was 1906 days (the patient represented on the furthest right of Figure 1); this was 1920 days after diagnosis. The longest that records were available for any patient i.e. from diagnosis up until the last day of contact, was 12,537 days.

The data were analysed independently of Caris. Patients were covered under 1 of 4 different protocols or exemptions, listed as follows. (1). The Caris Registry Protocol (TCREG-001-00-V2-1209) was approved by WIRB (WIRB Tracking #20092285) and has an NCT# of NCT02678754. (2). The Caris POA Prospective Repository (COE-001-0815) was approved by WIRB (WIRB Tracking #20162864) and has an NCT# of NCT03324841. (3). The Caris POA Retrospective Repository (COE-002-0116) was approved by WIRB (WIRB Tracking #20162657) and has an NCT# of NCT 00326499. (4). ION data is covered under an IRB exemption. All data are retrospective and have been de-identified prior to Caris receiving it and authors performing independent analyses.

**CONFLICTS OF INTEREST**

Authors received no funding or honoraria for this publication. The data were analysed independently of Caris. Thomas Herzog and Lee S. Schwartzberg are on the scientific advisory board of Caris Life Sciences.

Original article: Oncotarget. 2018; 9:9456-9467. https://doi.org/10.18632/oncotarget.24258

